# Address

**Published:** 1877-12

**Authors:** G. R. Thomas

**Affiliations:** President


### THE DENTAL REGISTER.

VOL. XXXI.] DECEMBER, 1877. [NO. 12.

### ADDRESS.

*Before the Michigan State Dental Society, Oct. 10th, 1877.*

BY THE PRESIDENT, DR. G. R. THOMAS.

Brothers of the Michigan State Dental Association: I am not aware of any thing in the constitution or by-laws of our association, that makes it obligatory upon the executive officer, to present a special address. But in order not to digress from what has been the custom of my predecessors, I shall detain you for a short time only, on a subject, that to me, is full of interest. These annual meetings are always occasions for joyful congratulations, a short respite from routine labor, and an interchange of thought upon questions vital to the growth of our profession.

It does us all good, to shake each other by the hand. As a profession we naturally pride ourselves on the rapid advances that have been made during the past ten or fifteen years, and I think none outside the profession will question our right to indulge in a little pride in that direction. But is it not well for us to stop occasionally, and ask ourselves if *all* this advance movement is in the direction of the "greatest good to the greatest number?" If it is, we are all ready, with one accord, every where, to join in a hearty amen. If it is not, then it becomes us to retrace some what and see if we can detect where changes beneficial to our patients can be inaugurated. While we have been feasting ourselves over the advent of the mallet, the rubber dam, the burring engine, cohesive gold, and kindred other good things for the preservation of the natural teeth, have we not been a little negligent in carrying forward what is called the strictly mechanical part of dentistry? I think none of us would have much trouble in forming an opinion on this subject, if we were to take promiscuously, ten young men from an average class of dental graduates, and compare the success that will attend their efforts for saving the natural teeth, and the work that will emanate from their laboratories to replace the lost, with the oral operations and laboratory work, of ten men, twenty or thirty years their seniors. In saving the natural teeth, I am inclined to the belief, that the seniors would be compelled to surrender to the juniors. But how about the skill displayed in the manufacture of artificial dentures? Are the young men of to-day, as well prepared to supply the lost teeth in as good and substantial a manner, as were the men of twenty or thirty years ago? I think not. Do not misunderstand me, my comparison refers to the average dentist of *then*, and *now*. If this is so, and I think you will all agree with me, there must be some potent and tangible reason for it. Is it necessary that while one branch of dentistry is going forward, the other is retrograding? This introduces the question, are there separate and distinct departments in dentistry? Undoubtedly there are; and always will be.

Then comes the question, does the dentist, who prepares himself for, and carries forward a general practice, serve his patients as well, as the practitioner of a special department?

We find in all the professions, I think, that a majority attempt to carry forward a general practice in whatever profession they may be engaged, and yet, if you or I have the misfortune to have a dear friend stricken with an unyielding and dangerous disease, our first and only thought is to employ a physician who makes that disease a specialty. If we have a case at law where great interests are at stake, we employ a man who makes that branch of the law, that covers our case a specialty, and so it is in all professions, and trades, which proves that the better a man prepares himself for any one specialty, and practices it, the better does he serve his patrons.

And now, as to the reason for the retrogression of one department of dentistry, while the other has advanced so rapidly.

On the one hand, the introduction of vulcanite has made it possible for men to make *themselves* believe, and the public for a time, that from three to six months pupilage makes a dentist, and this of itself has been enough to drag the mechanical department *down*, DOWN, DOWN, and the operative as well, in the hands of such men. While on the other hand, the rapid advance made in the manufacture of appliances for saving the natural teeth, together with the greatly increasing demand for the same, has been a decided stimulus for the best men in the profession, to turn all their energies in that direction.

And nothing is more natural. It is difficult to keep a man sawing wood at a dollar a day, when he finds he can make five dollars a day running a steam engine;, still, there always will be plenty of men who will saw wood for a dollar a day, while men are making five dollars a day running steam engines. But the same man will not be likely to engage in the sawing of wood, and the running of an engine.

Has this unequal state of education been a misfortune to the profession? I doubt not the majority of dentists, without reflection, would answer, *yes*.

I am beginning to think not, but that much good will come out of it. Had the same thorough training been carried forward in the mechanical department, as in former years, could the operative department have been brought to that standard of perfection that it now enjoys?

Is it possible for the same genius to excel in two directions that are so diverse, as is operating in the mouths of nervous and delicate individuals, over the most sensitive tissues, requiring a delicacy of touch, that only the best trained fingers can lay claim to, meeting disease in every form that the oral cavity presents, as well as the sympathetic diseases of the whole body, and, the hot blasts from the melting pots, the blow pipe, and the continuous gum furnace, the moulding sand, the wax and plaster knives, the melting and refining, the anvil and lathe, the vulcanizer and scrapers, and all the paraphernalia necessary to carry forward the work of a well appointed laboratory?

I ask, is it possible for one person to *excel* in all this? Suppose the education is sufficient in both directions, are the *hands* that are engaged in the manufacture of artificial dentures, in as fit condition to operate over the delicate tissues of the oral cavity, as are those that know nothing of the workings of the laboratory? In answer to this, some will say, my laboratory work is done by an assistant, my fingers are not soiled over plate work. But who is that assistant? Is he a man of experience in the laboratory, and competent to do all the work necessary to produce the best results? If so, then he should have free access to, and full charge of the patient during the time his work is going on; but is he not more likely to be a student, put into the laboratory more for the sake of getting *him* out of the way,—as well as the dirty work—than for the production of first class plate work? Can first class plate work be produced in any such way? A great many dentists give their plate work out to laboratory workmen to do, making the model, and taking the bite themselves, perhaps selecting the teeth also, but leaving all the rest to the laboratory workman. I do not believe the *best* results can be produced in this way. The man who arranges the teeth should have the patient with him. If every office could be supplied with two good men, one for each department, each thoroughly versed in his business, and alike interested financially, *then* I believe the *very best* results might be attained, and this brings us to the consideration of the education of the two men. Would it be necessary, or advisable for the man who is to make the best record possible, in saving the natural teeth, to spend his time and means to fit himself for the detail of laboratory work, that he never expects to follow? or, for the man who expects to make the production of artificial dentures his business, to spend his time to acquaint himself with the best means to cure disease, arrest decay, and perform the necessary operations that the oral surgeon is called upon for?

In all successful occupations, we find the labor divided. Go into any of our manufacturing establishments, and how often do you find one man *only*, producing the entire article? *very seldom*, and why is it? simply because the less detail and change there is in a man's labor, the better are his fingers prepared for the work. Take for instance the manufacture of so small an article as the pin, I am told that in the best regulated pin manufacturing establishments, there are no less than eight persons engaged in its production. I would not suggest however that more than one man should be engaged in the putting together of the various substances that compose an artificial denture. I believe I have never as yet expressed myself publicly as being decidedly in favor of the divorcement of the relation that has so long existed between operative and mechanical dentisty. But there are some seemingly vexed questions that are constantly under discussion, wherever and whenever dentists congregate. Whether we are a *factor* of the medical profession, or whether we are a distinct profession, is a question, both sides of which, are advocated by the very best men in the profession. It is very evident that if this divorcement ever really takes place, there will no longer be a question here; for men who are prepared, and engaged *only* in the treatment of the oral cavity, will have the same claim to medical recognition, as the man who is engaged in the treatment of the eye and ear, or any other part of the body; but just so long as we allow "three months" rubber men, to attach themselves to, and sail under the same flag, as the educated dental surgeon, we must expect to answer to the appellation of "tooth carpenters."

Most of you remember well, the unsuccessful efforts that have been made in this State, to procure the passage of a law to regulate the practice of dentistry. You remember too with a feeling of chagrin, some of the arguments that have been used to defeat its passage, by representatives in our legislature, of which the following is a sample in substance. I think it was in 1870 when a representative from Kalamazoo took the position, that if these dentists should succeed in getting a law passed, to regulate their work, it would not be long till the shoemakers, the blacksmiths, the stone masons, and kindred other tradesmen would petition for similar favors. And you remember too the criticisms that were made by the best dentists in this State, upon the use of such arguments, to crush one of the *best* sanitary measures that ever went before the legislature. Neither have you forgotten who were the sympathizers with the legislators who made use of such arguments. The men calling themselves dentists, whose chief occupation is the manufacture of cheap plate work, and plastic fillings. Those legislators were censured but did'nt they have an argument there that was potent? If the true oral surgeon could cut loose from such, would such arguments hold good for a moment? The fact is, plate work, is to-day being almost entirely neglected by the best men of the profession; and why is it? because it is not compatible with dental surgery. In our associations, mechanical dentistry is almost completely ignored. I think at the meeting of the American Dental Association at Chicago last August, there was one paper presented upon that subject, which was passed with very little if any discussion whatever. Examine the announcements of our State, and local societies, and more than likely mechanical dentistry has no place whatever among the standing committees. Go into the offices of the best dentists throughout the land, and half of them will have no laboratories, while the other half will have only apologies for them, all of which goes to show that separation is not so far off as some would have it; and I am of the opinion that great good will eventually come out of it.

The waters of the riled fountain will work themselves clear the sediment always disappearing at the bottom. Just here some one may ask, "What! would you have plate work go entirely into the hands of cheap Johns and Quacks?" By no means, there is no reason why mechanical dentistry cannot be made of itself, as noble, and honorable a calling, as is operative dentistry; and the way to do it, is to train men especially for it, and it alone. But as at present conducted in most of the colleges, a young man becomes anxious to get to operating almost as soon as he enters, and *reluctantly* learns just enough of laboratory work to pass a formal examination. I am glad to know however, that we have some good and noble men in the country who are not ashamed to say that they are mechanical dentists exclusively; whose work would put to shame the laboratory efforts of some of our acknowledged leading dentists, who attempt to practice in both departments. Such a man is Dr. L. P. Haskell of Chicago, who has made mechanical dentistry a specialty for nearly thirty years. Dr. Keith of St. Louis, of whose acquaintance I have had the pleasure a few days since in our own city, is making mechanical dentistry, a specialty. Dr. W. L. Willet of the firm of Wilcox & Willet of Newark, New York, I understand devotes his entire attention to mechanical dentistry, while his partner attends wholly to the operative department; and it seems to me, that this last mode of practice, is the most practical way out of the present difficulty; for I am well aware, that one of the strong arguments in opposition to this divorcement, is, that if the mechanical department is left wholly for men to make a specialty of, they will naturally be so anxious to enlarge their business, that they will sacrifice good teeth, in order to acomplish it; (I would confine the extraction of teeth to the dental surgeon, always,) or that they will turn their hands at various times to operating upon the natural teeth, producing inferior operations. The public however, will soon learn to distrust such men, if the separation ever really takes place. Some will make the argument that the plan might be made to work in cities, "but how about the country!" At the present time there are very few towns so small that they do not support two dentists at least; and most of them more; some few however, only one, and as at present conducted each man is engaged partly at plate work, and partly at chair work. I am of the opinion that there is no one thing that would tend to bring about a harmonious action, and fraternal feeling among dentists, so quickly, and so effectually, as a division of this kind, in *cities* and in *towns*, *everywhere*; and I am convinced, that in canvassing this matter a little, we would be surprised at the number of men who would perfer to do plate work only. I believe that if the schools would adopt the plan of teaching separately, and granting separate diplomas, to mechanical dentists, and oral surgeons, that a great step in the right direction would be taken.

I do not look for any great stampede in the direction of its accomplishment; if it ever comes about as a recognized fact, it will be by men drifting into that department which pleases them best, and by a laudable ambition to excel in the department chosen, until the schools will be called upon to teach separately the two departments, and grant diplomas accordingly. I may be asked, "If you favor this movement, why do you not adopt it at once? I do not expect every one who favors it, to adopt it. Most of us, if not all, have started out in practice, by doing plate work for our patients, and now they expect, and demand it of us, and it seems quite impossible for us to drop it at once, without we know of some person in our own locality who makes that a specialty and will serve them well; and this seems to me, to be the one thing lacking in most localities: good, competent, exclusively, mechanical dentists. If I was to start again, with my present experience, I certainly would practice in one department only, for this reason: I believe that in so doing I could render more valuable service to those whom I served.

And now gentlemen, allow me to express to you my sincere thanks for the honor you have the second time conferred upon me, in entrusting the executive office of this association to my care, for I know it is not because of any particular merit, or fitness that I possess, but a mark of courtesy, and it is for that reason I value the honor. The past year has been marked by no particular line of advancement in the profession, as far as I am aware, except the replanting of teeth, which seems to have more than satisfied the expectations of those who have practiced it to any extent. To me, it is a mode of practice that opens up an entirely new field, where we can bestow unlimited benefit upon our patients; for the benefits to be derived are mostly confined to those who are suffering with teeth that are almost, if not quite past being operated upon in the mouth, for which the remedy heretofore has largely been extraction. Since one year ago last April, I have operated upon more than fifty cases, without the loss of a tooth yet, so far as I know, except one (which I do not regard as a failure on account of the operation.) It was a large molar for a gentleman about forty years of age, and being quite unaccustomed to pain, he returned to the office in less than two hours from the time the operation was performed, declaring that he could not and would not, endure the pain longer; I consequently extracted the tooth again, being none the less satisfied however, that had he borne with it for a short time longer (for I had known the same thing to occur many times before) an entirely satisfactory result would have followed.

I cannot close without a word in reference to our Dental College. The members of this association naturally feel a just pride in its success, many of them having been its projectors and fosterers, and I am sure they feel well repaid for the anxiety and care they have thus far bestowed upon it. Our beginnings were small, our appropriation was limited to $3,000,00 a year for the first two years; but I believe we are all well assured that it was economically, and judiciously expended. The number of matriculants in our first class was twenty, of whom nine graduated at the first commencement; the number of matriculants for the second year was thirty-three, of whom ten graduated. The class is now in the beginning of the second week with thirty-nine matriculants, which is evidence of a steady and healthy growth, and we believe, with good management in the future, the success of the institution, is beyond the experimental border. The appropriations, by the last legislature, were increased to $6,000,co a year. We think we could have used more to good advantage, but in view of the generally depressed financial condition of the country, and the disposition manifested for general economy, we do not complain of the meager amount furnished. But I do feel hopeful that the showing this department will be able to make, by the time the next legislature convenes, will be such, that our legislators, will not only be willing to grant a sufficient appropriation for the full maintenance of the department, fully equipped, but will be actuated by a sense of justice to pass a bill, that will in a measure protect that class of our citizens,—who never make much inquiry as to a man's fitness,—from quackery and charlatanism. And I have no doubt the passage of such a bill can be secured, by proper and prompt action on the part of the members of this association.

